# A Study on Parental Corticophobia in Pediatric Allergic Diseases

**DOI:** 10.3390/medicina61111959

**Published:** 2025-10-31

**Authors:** Halil Alkaya, Uğur Altaş, Seda Çevik, Yakup Söğütlü, Mehmet Yaşar Özkars

**Affiliations:** 1 Department of Pediatric Allergy and Immunology, Ümraniye Training and Research Hospital, İstanbul 34760, Türkiye; 2 Department of Pediatric Emergency Medicine, Ümraniye Training and Research Hospital, University of Health Sciences, İstanbul 34760, Türkiye

**Keywords:** parental beliefs, corticophobia, pediatric allergy, prevalence, childhood asthma, allergic rhinitis, atopic dermatitis, health education, family-centered care, information needs, associated factors

## Abstract

*Background and Objectives*: Parental beliefs strongly influence treatment adherence in pediatric allergic diseases. Concerns about corticosteroid therapy—known as corticophobia—may disrupt disease control and compromise child well-being. This study aimed to evaluate parental knowledge, beliefs, and concerns regarding topical, inhaled, and intranasal corticosteroid use in children, and to identify sociodemographic factors associated with corticophobia. *Materials and Methods*: This prospective survey was conducted in a tertiary pediatric allergy and immunology clinic. A structured questionnaire was anonymously completed by 110 parents of children receiving corticosteroid therapy. The survey assessed demographics, family history of atopy, corticosteroid use, perceived disease severity, knowledge level, concerns, and sources of information. Descriptive statistics and chi-square tests were applied (*p* < 0.05 significant). *Results*: The most frequent concerns were growth retardation, hormonal imbalance, and long-term side effects. Corticophobia was significantly more prevalent among university-educated parents (*p* = 0.043) and those with a family history of atopy (*p* = 0.017). Despite generally high adherence to prescribed regimens, nearly 60% of parents sought additional information, highlighting the impact of knowledge gaps on health-related parenting practices. *Conclusions*: Corticophobia remains a common parental concern in pediatric allergy care, with implications for adherence, family decision-making, and child well-being. Addressing misinformation and providing family-centered, tailored educational strategies—particularly for highly educated parents and those with an atopic background—may reduce fears, strengthen trust, and promote sustainable healthy behaviors.

## 1. Introduction

Glucocorticoids have been used as therapeutic agents in the treatment of various inflammatory diseases since their discovery over 60 years ago. Although they are safe, effective, and inexpensive, healthcare professionals may hesitate to prescribe corticosteroids due to parental concerns and misconceptions about these treatments. Glucocorticoids are derived from cortisol, a significant glucocorticoid hormone [1,2].

Systemic cortisol plays a key role in metabolic regulation and must be replaced when insufficiently produced by the adrenal glands. Topical glucocorticoids suppress inflammatory pathways and have the potential to inhibit cortisol production [3]. When applied to multiple areas of the body simultaneously, used over prolonged periods, or applied frequently, their absorption through the skin may increase [4]. Factors such as humidity and temperature can amplify the effects of these medications on the skin, and many patients respond well to treatment [5].

Glucocorticoids can increase anti-inflammatory cytokines, induce eosinophil apoptosis, slow leukocyte function, and reduce mast cell sensitivity. However, they may also lead to metabolic effects such as elevated growth hormone levels, increased bone resorption, elevated blood glucose, and heightened protein catabolism [3,6,7]. Topical corticosteroids may cause side effects such as skin thinning (atrophy), excessive hair growth (hirsutism), delayed wound healing, acne, perioral dermatitis, tinea incognito, photosensitivity, skin dryness and cracking, telangiectasia, and purpura. Guidelines recommend the use of very potent glucocorticoids only during acute flare-ups on the palms and soles [8,9]. Most side effects are reversible and can be detected early through close monitoring [10,11].

Inhaled corticosteroids (ICS) are fundamental in asthma treatment and reduce disease-related morbidity and mortality [12]. Traditionally, ICS were used when severe attacks persisted, symptoms increased in frequency, or pulmonary function tests declined [13]. When used regularly in therapeutic doses, they are not expected to cause growth retardation. Uncontrolled asthma is common, with a reported prevalence of 40–53% among patients with moderate-to-severe asthma [14,15]. In cases in which asthma control is not achieved with ICS, oral corticosteroids (OCS) are added. However, it remains unclear whether low-dose use leads to adrenal suppression [16]. Short-term use of ICS has been shown to be effective and safe. Nonetheless, considering that the SMART (Single Maintenance and Reliever Therapy) strategy represents a continuous treatment approach, it should be noted that inhaled ICS may be required to maintain symptom control as needed [17]. Their regular use is now practiced to control asthma and when used in the suggested therapeutic doses, they are not expected to cause impaired growth. Nasal corticosteroids are effective and safe during childhood [18]. Although nasal irritation and epistaxis are occasionally observed during intranasal corticosteroid use, their incidence remains below 10%, and septal perforation is an extremely rare side effect. Additionally, growth retardation is a rare occurrence. When applied correctly, these medications do not lead to serious adverse effects [19].

The primary reasons for parents’ skepticism toward corticosteroid therapy are lack of knowledge and concerns regarding potential side effects. In pediatric care, parental beliefs, health literacy, and attitudes toward long-term treatments have a direct impact on children’s adherence, disease control, and overall development. In addition, factors such as the child’s refusal to take the medication and physicians modifying or discontinuing treatment further contribute to parental hesitations [20].

Concerns surrounding corticosteroid use are commonly referred to as “steroid phobia.” In a previous study involving patients with atopic dermatitis, 80.7% expressed anxiety about corticosteroid use, and 36.7% reported reluctance to initiate or continue treatment. Factors such as prior negative experiences, uncertainty regarding proper dosage, and the desire to discontinue medication quickly have all been associated with this phenomenon. However, when used appropriately, corticosteroids remain a safe and effective cornerstone in the management of inflammatory pediatric conditions.

Parental attitudes toward various corticosteroid formulations—particularly topical, inhaled, and intranasal forms—play a critical role in treatment adherence in children with asthma, allergic rhinitis, and atopic dermatitis. Misconceptions regarding systemic effects, such as growth suppression and adrenal insufficiency with inhaled corticosteroids, or local side effects such as nasal irritation and epistaxis with intranasal formulations, may contribute to varying levels of parental concern. These concerns, often rooted in inadequate or inaccurate information, can lead to resistance to treatment, ultimately compromising disease control and reducing the child’s quality of life.

This study aims to evaluate parents’ knowledge, concerns, and sources of information regarding different corticosteroid delivery routes, and to analyze how these perceptions correlate with demographic characteristics, parental roles in healthcare decisions, and the presence of a family history of atopic disease. The findings are intended to inform more targeted educational strategies, strengthen physician–parent collaboration, and improve treatment adherence, thereby supporting optimal health and developmental outcomes in pediatric patients.

## 2. Materials and Methods

### 2.1. Study Design and Setting

This prospective, cross-sectional survey study was conducted to evaluate corticosteroid hesitancy among parents of children diagnosed with pediatric allergic diseases, including asthma, allergic rhinitis, and atopic dermatitis. Participants were consecutively recruited from the Pediatric Allergy and Immunology Outpatient Clinic of Ümraniye Training and Research Hospital, a tertiary referral center, between September 2024 and December 2024.

### 2.2. Questionnaire Development

The structured questionnaire was developed based on previously validated instruments assessing corticosteroid phobia and treatment adherence in pediatric allergy and dermatology settings, including the studies by Aubert-Wastiaux et al. (2011) on topical corticosteroid phobia [9] and Özçeker et al. (2018) on parental corticosteroid concerns in asthma [12]. Relevant items were adapted from these instruments and refined for cultural and linguistic appropriateness. Content validity was established through expert review by two pediatric allergists and one clinical psychologist. A pilot test with 15 parents was conducted to ensure clarity and comprehension, and minor modifications were made accordingly. In this study, corticophobia was defined as parental fear or hesitation related to corticosteroid use, encompassing both concerns about the treatment itself (e.g., drug dependence, long-term use) and worries about potential side effects (e.g., growth retardation, hormonal imbalance, obesity). This operational definition was adapted from prior studies on corticosteroid phobia [9,12] and was assessed through questionnaire items included in the “Parental Knowledge and Concerns” section.

### 2.3. Questionnaire Structure

The final questionnaire consisted of four main sections with a total of 32 items:

1. Demographic Information (7 items): Parent’s age, gender, educational level, socioeconomic indicators, and child’s age, gender, and diagnosis.
2. Clinical Characteristics (6 items): Type of allergic disease, corticosteroid formulation used (topical, inhaled, nasal), duration of use, frequency of use, and prior discontinuation history.
3. Parental Knowledge and Concerns (12 items): Items addressing awareness of potential side effects (e.g., growth retardation, hormonal imbalance, obesity), perceived disease severity (0–10 scale), and overall confidence in corticosteroid therapy.
4. Sources of Information (7 items): Questions about where parents obtained information (physician, internet, family/friends, social media, written leaflets).

Response formats included Likert-type scales (1–5 from strongly disagree to strongly agree), Yes/No options, and multiple-choice items, as well as one open-ended question to capture additional concerns.

### 2.4. Participants and Recruitment

Eligible participants were parents of children aged 0–18 years who had been prescribed corticosteroid therapy for at least 1 month (inhaled), 2 weeks (nasal), or 1 week (topical). Parents were approached in the outpatient clinic waiting area, informed about the study, and those who provided written informed consent were included. Parents of children who had used corticosteroids below these thresholds or who declined participation were excluded.

### 2.5. Sample Size Justification

On average, approximately 120–150 parents visit the Pediatric Allergy and Immunology outpatient clinic per month. During the three-month study period, approximately 400–450 eligible parents were expected. Recruitment using convenience sampling continued until 110 fully completed questionnaires were obtained.

The target sample size was estimated using power analysis for a chi-square test, with a medium effect size (w = 0.3), alpha = 0.05, and power = 0.80, which yielded a minimum required sample of 88 participants. The inclusion of 110 parents therefore exceeded the required sample size, allowing for robust statistical analysis despite the single-center design.

### 2.6. Data Collection

To minimize social desirability bias, questionnaires were completed anonymously in a separate area under the supervision of an independent researcher unaffiliated with the clinical team.

### 2.7. Statistical Analysis

All analyses were performed using IBM SPSS Statistics for Windows, Version 26.0 (IBM Corp., Armonk, NY, USA). Normality of continuous variables was assessed with the Kolmogorov–Smirnov test. Since most variables did not follow a normal distribution, continuous data were summarized as medians and interquartile ranges, and categorical variables as frequencies and percentages. Between-group comparisons were performed using the chi-square test or Fisher’s exact test, as appropriate. Likert-scale responses were treated as ordinal variables. No multivariable analysis was performed due to the limited sample size. A two-tailed *p*-value of <0.05 was considered statistically significant.

- Ethics

The study was approved by the Ethics Committee of Ümraniye Training and Research Hospital (Decision No: 258, Date: 5 September 2024). Written informed consent was obtained from all participating parents.

- Data Availability Statement

The data supporting the findings of this study are available from the corresponding author upon reasonable request.

- GenAI Use Disclosure

No generative artificial intelligence (GenAI) tools were used for data collection, statistical analysis, figure creation, or manuscript writing in this study.

## 3. Results

### 3.1. Demographics

A total of 110 parents participated in the survey. The median age of the parents was 34 years, while the median age of the children was 4 years. Of the children, 50.9% (*n* = 56) were male. As shown in Table 1, most respondents were mothers (84.5%), and 40% (*n* = 44) of the parents were high school graduates. Overall, the majority had completed high school or higher education. Regarding diagnoses, 40.9% (*n* = 45) of the children had asthma, 31.8% (*n* = 35) had allergic rhinitis, and 27.3% (*n* = 30) had atopic dermatitis. A family history of atopic disease among parents or first-degree relatives was reported in 60.9% (*n* = 67) of participants.

### 3.2. Knowledge and Perceptions

**Figure 1:**
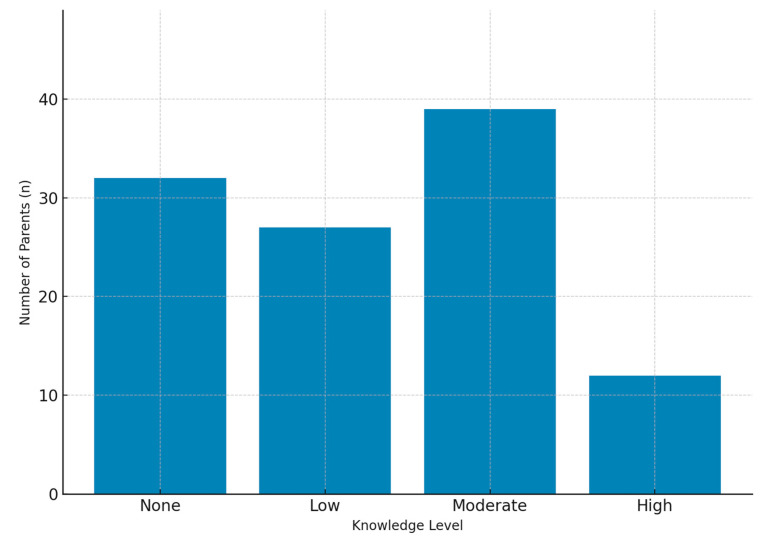
Perceived Knowledge Level and Disease Severity Among Parents. Parental knowledge levels are expressed as percentages. Disease severity was rated on a 0–10 scale by each respondent.

As shown in Figure 1, parental knowledge levels and perceived disease severity were evaluated. Knowledge levels are displayed as percentages, while disease severity was rated on a 0–10 scale.

Knowledge levels are displayed as percentages of parents reporting ‘no knowledge,’ ‘low knowledge,’ ‘moderate knowledge,’ or ‘very well-informed. This figure illustrates both the distribution of parental knowledge and the overall perception of disease severity.

### 3.3. Statistical Associations

No significant associations were found between corticosteroid-related fears and parental gender, perceived disease severity, the reassuring effect of prescription information, or the parent’s self-reported knowledge level. However, fear rates were significantly higher among university-educated parents (72.7%, *n* = 24) compared to those with a high school education or less (51.9%, *n* = 40) (*p* = 0.043) (Table 2).

Corticophobia was significantly more prevalent among university-educated parents compared to those with a lower educational background (72.7% vs. 51.9%, *p* = 0.043). Similarly, parents with a family history of atopy reported higher rates of corticophobia than those without such a history (67.2% vs. 44.2%, *p* = 0.017). These findings suggest that both parental education and familial atopic background are key factors associated with increased corticosteroid-related fears.

### 3.4. Parental Concerns

**Figure 2:**
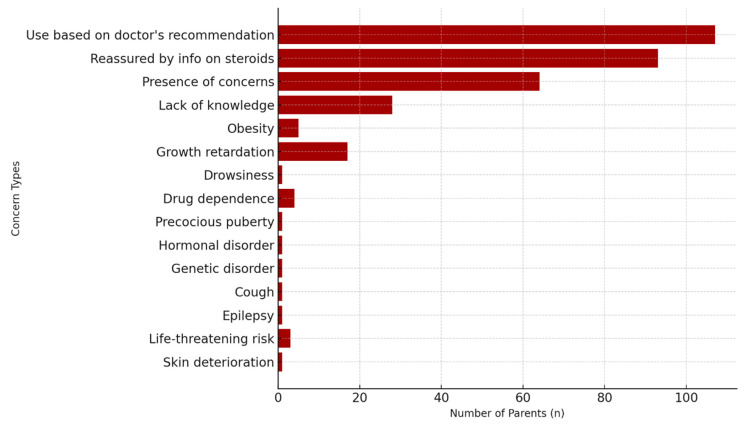
Parental Concerns and Reasons for Concern About Steroids. Frequencies of specific concerns reported by parents are shown, including growth retardation, obesity, hormonal disturbances, drug dependence, and other less frequent issues such as cough, epilepsy, genetic conditions, and skin changes.

A vast majority of parents (97.3%, *n* = 107) reported using corticosteroid medications based on their physician’s recommendation. Most parents (84.2%, *n* = 93) indicated that the information provided about these medications was reassuring. Nevertheless, more than half (58.2%, *n* = 64) still reported ongoing concerns. The most frequently cited reason was a lack of knowledge (43.8%, *n* = 28), followed by growth retardation (26.6%, *n* = 17), obesity (7.8%, *n* = 5), and drug dependence (6.3%, *n* = 4). Less common concerns included drowsiness, precocious puberty, hormonal imbalance, genetic disorders, cough, epilepsy, and skin deterioration, each reported by one parent. These findings suggest that despite basic counseling, many parents still require deeper, more targeted education about corticosteroid use and safety (Figure 2).

## 4. Discussion

In our study, we assessed parents’ knowledge, beliefs, and concerns regarding the use of corticosteroids delivered via topical, intranasal, and inhaled routes in their children. Among the children, 40.9% (*n* = 45) had asthma, 31.8% (*n* = 35) had allergic rhinitis, and 27.3% (*n* = 30) had atopic dermatitis. In the literature, research on corticophobia (steroid phobia) has largely focused on patients with atopic dermatitis and the use of topical corticosteroids [21,22]. Our study, by contrast, aimed to provide a broader perspective and contribute novel insights to the field.

The median parental age was 34 years, which is comparable to the findings of Göksügür et al., who also reported a mean parental age of 34 years [22]. Parental age is thought to influence knowledge levels and concerns regarding corticosteroid use in children. Older parents may have higher health literacy and thus be more informed about corticosteroids, whereas younger parents may be more prone to concerns about potential side effects and adopt a more cautious approach to treatment. Although our study did not stratify participants by age or explore age-related differences in detail, future studies should examine this variable more thoroughly. Investigating the relationship between parental age, knowledge, and concerns would enable the design of targeted age-specific educational interventions.

The association between educational level and concerns regarding corticosteroid use is noteworthy. Parents with higher educational attainment may have a better understanding of potential side effects; however, this awareness can sometimes result in more critical or cautious attitudes toward treatment. In contrast, parents with lower educational backgrounds may be more inclined to trust physicians’ recommendations during the course of therapy. Therefore, it is important that scientific information be communicated clearly and in an accessible way to parents across all educational levels. Providing accurate and easy-to-understand information about corticosteroids can help strengthen trust and reduce unnecessary fears. Further studies are needed to evaluate the effectiveness of educational strategies designed for parents with different educational backgrounds. In addition, offering parents personalized information is crucial to enhance their confidence in healthcare professionals and to minimize misconceptions about corticosteroid use.

A family history of atopy was present in 60.9% (*n* = 67) of participants. Among these, 67.2% of parents reported concerns about corticosteroid use, compared with 44.2% of those without a family history of atopy (*p* = 0.017). These findings suggest that personal or familial experiences with allergic diseases may amplify concerns and highlight the importance of considering family history during corticosteroid prescription. Providing detailed counseling to parents with an atopic background may be particularly beneficial. However, the current evidence base is limited, and larger, multi-population studies are warranted to further clarify this association.

Although most parents reported trusting their physicians’ recommendations, a considerable proportion also expressed the need for more detailed information. This finding suggests that parental perceptions of treatment can be shaped significantly by the quality of communication and counseling provided. Previous work has shown that sensitive, thorough pre-treatment discussions can substantially reduce corticophobia [23]. In our study, 97.3% of participants reported using corticosteroids based on medical advice, and 84.2% found the information provided reassuring. Nevertheless, nearly 80% indicated a need for additional knowledge, underscoring the importance of comprehensive and transparent education during clinical care. In addition to physicians, pharmacists—who are globally regarded as accessible and trusted healthcare professionals—can also play a critical role by providing detailed information about proper medication use, potential side effects, and safe treatment practices, thereby reinforcing adherence and reducing parental fears.

The most common concerns expressed were insufficient knowledge, growth retardation, and the risk of obesity. Endocrine side effects such as impaired growth and weight gain have been consistently reported as major parental concerns in prior studies [9]. Our findings further revealed frequent worries about hormonal disturbances and drug dependence. Interestingly, some parents also reported concerns unrelated to corticosteroid use—such as cough, epilepsy, or genetic disorders—highlighting the persistence of misinformation and misconceptions. The fact that a proportion of parents admitted lacking adequate knowledge about corticosteroids further emphasizes the need for improved education by healthcare professionals. Targeted educational programs correcting misconceptions could improve adherence and reduce unnecessary fears.

Accurate information can enhance parental trust. Physicians should therefore provide clear, reassuring explanations regarding both the risks and benefits of corticosteroid use, addressing widespread misconceptions in a direct yet empathetic manner. Even though treatment adherence was generally high, enhancing the quality of adherence—ensuring correct dosing and duration—is crucial. Misinformation-driven practices, even when superficially aligned with physician advice, may still contribute to anxiety, hesitation, or misuse. Strengthening parental education is therefore essential to optimize treatment outcomes and improve children’s quality of life.

Notably, parents who expressed a need for additional information were significantly more likely to exhibit corticophobia (*p* = 0.005). This finding highlights the impact of knowledge gaps as a major factor contributing to treatment-related fears and emphasizes the importance of targeted education. One study reported that nearly one-third of participants obtained misinformation about corticosteroids from family members, friends, or online sources [24]. Given the prevalence of such misinformation, it is understandable that parental concerns remain persistent. Clear communication and well-structured educational strategies are therefore essential to reduce anxiety and build greater trust in therapy.

In recent years, advances in corticosteroid formulations and treatment approaches may also have influenced parental perceptions. The introduction of newer intranasal and inhaled corticosteroid preparations with improved safety profiles and shorter treatment durations has been suggested to alleviate long-term concerns about adverse effects [25]. Recent studies further support these observations, showing that parental fears and knowledge deficits remain widespread across different cultural settings [26,27,28,29]. For instance, one nationwide cross-sectional study [26] reported that a large proportion of parents exhibited corticosteroid-related fears and knowledge gaps, whereas another study demonstrated that cultural factors may influence parental attitudes [27]. Other investigations have noted that parents’ prior experiences and social environments strongly shape their perceptions of topical corticosteroid use [28,29]. Evidence published within the past five years continues to reinforce these findings [30].

Most prior studies on corticophobia have been restricted to atopic dermatitis and topical corticosteroid use, often neglecting other common pediatric allergic conditions in which corticosteroids represent a cornerstone of treatment. By contrast, our study expands this perspective by simultaneously investigating inhaled, nasal, and topical corticosteroids in children with asthma, allergic rhinitis, and atopic dermatitis. This broader scope provides a more realistic reflection of pediatric allergy practice and offers a more comprehensive assessment of how parental concerns influence treatment adherence across multiple allergic diseases.

Furthermore, our study is among the few to analyze the impact of sociodemographic variables—particularly parental education level and family history of atopy—on corticophobia. We also identified specific misconceptions, ranging from growth retardation and obesity to unrelated concerns such as epilepsy or genetic disorders, which further illustrate the role of misinformation in shaping parental fears. These contributions fill a notable gap in the literature by moving beyond disease-specific or treatment-route–specific analyses, highlighting cross-cutting parental perceptions, and defining high-risk groups that may benefit most from tailored educational interventions.

Adherence to corticosteroid therapy is a key factor in achieving effective and lasting treatment outcomes. Educational programs that highlight the safe use of corticosteroids, ways to minimize side effects, and the overall benefits of therapy can help strengthen parents’ confidence in treatment. In this context, one study identified several predictors of parental adherence and emphasized that well-designed educational interventions may enhance treatment success [29]. For families with higher educational levels or an atopic background, providing more detailed and reassuring information may be particularly effective in reducing anxiety and improving adherence. Future studies should explore the prevalence and impact of corticophobia across different age groups and socioeconomic settings. In addition, randomized controlled trials assessing the long-term effects of educational interventions could help refine strategies to address parental concerns and ultimately improve both adherence and quality of life in children with chronic allergic diseases.

### Limitations

This study has several limitations. First, data on income level were not collected, and socioeconomic status could not be assessed. The study included only topical, inhaled, and intranasal corticosteroid users, while oral corticosteroid use was excluded. In addition, the age of onset of the allergic disease and treatment-related benefits were not evaluated. The relatively small sample size and use of a convenience sampling method may limit the generalizability of the findings, and the single-center design may not fully reflect the diversity of parental beliefs in other regions or healthcare settings. Furthermore, potential confounding variables—such as socioeconomic status, parental age, previous healthcare experiences, and physician–parent communication styles—were not fully controlled and may have influenced attitudes. Finally, the cross-sectional design precludes causal inference between corticophobia and treatment adherence. Future multicenter studies with larger, more diverse samples and longitudinal designs are warranted to validate and extend these results.

## 5. Conclusions

This study demonstrates that parental corticophobia remains a prevalent concern in the management of childhood allergic diseases, even when adherence to prescribed corticosteroid regimens appears high. Our findings highlight that higher parental education and a family history of atopy are associated with greater fear, emphasizing the complex interplay between knowledge, prior experiences, and treatment perceptions. Beyond common concerns such as growth retardation and hormonal disturbances, the persistence of misconceptions—including fears unrelated to corticosteroid use—reflects the ongoing influence of misinformation.

Addressing these concerns requires more than routine counseling: tailored, family-centered educational strategies that adapt to sociodemographic backgrounds are crucial. Empowering both parents and families with clear, accessible, and evidence-based information can reduce unnecessary fears, foster trust in healthcare providers, and support sustainable adherence. Ultimately, integrating structured educational interventions into routine allergy care may improve treatment outcomes and enhance the quality of life for children with asthma, allergic rhinitis, and atopic dermatitis.

## Figures and Tables

**Table 1:** Sociodemographic Characteristics and Clinical Features of the Patient.

		*n*	%
Parent	Mother	93	84.5
	Father	17	15.5
Child’s Gender	Female	54	49.1
	Male	56	50.9
Parent’s Education Level	Primary School	20	18.2
	Middle School	13	11.8
	High School	44	40.0
	University	31	28.2
	Graduate	2	1.8
Diagnosis	Asthma	45	40.9
	Allergic Rhinitis	35	31.8
	Atopic Dermatitis	30	27.3
Family History of Atopy	Yes	67	60.9
	No	43	39.1

Atopic diseases included asthma, allergic rhinitis, and atopic dermatitis. Parental education categorized from primary school to graduate level.

**Table 2:** Factors Associated with Concern About Steroids.

		Presence of Concern About Steroids				
		No Concern		Concern		
		*n*	%	*n*	%	
Parent	Mother	37	39.8%	56	60.2%	0.312
	Father	9	52.9%	8	47.1%	
Education Level	University and above	9	27.3%	24	72.7%	0.043
	High school and below	37	48.1%	40	51.9%	
Family History of Atopy	No	24	55.8%	19	44.2%	0.017
	Yes	22	32.8%	45	67.2%	
Perceived Disease Severity	<7	27	49.1%	28	50.9%	0.122
	≥7	19	34.5%	36	65.5%	
Did medication information provide reassurance	No	7	58.3%	5	41.7%	0.352
	Yes	28	43.8%	36	56.3%	
Need for more information about medication	No	15	68.2%	7	31.8%	0.005
	Yes	31	35.2%	57	64.8%	
Perceived steroid knowledge level	None/Low	27	45.8%	32	54.2%	0.367
	Moderate/High	19	37.3%	32	62.7%	

## Data Availability

The raw data supporting the findings of this study are available from the corresponding author upon reasonable request.
